# Suppression of STK11 induces expansion of polymorphonuclear myeloid-derived suppressive cells and activation of immune signaling in breast cancer

**DOI:** 10.1007/s00262-025-04189-8

**Published:** 2025-10-06

**Authors:** Tzu-Hui Wei, Chi-Che Hsieh, Zhu-Jun Loh, Wei-Pang Chung, Kuo-Ting Lee, Yi-Ling Chen, Hui-Ping Hsu, Che-Hung Shen

**Affiliations:** 1 Department of Surgery, National Cheng Kung University Hospital, College of Medicine, National Cheng Kung University, No. 138 Sheng-Li Rd, Tainan City, 704302 Taiwan; 2 School of Dentistry, College of Oral Medicine, Taipei Medical University, Taipei, 11031 Taiwan; 3 Department of Oncology, National Cheng Kung University Hospital, College of Medicine, National Cheng Kung University, Tainan, 704302 Taiwan; 4 Center of Applied Nanomedicine, College of Medicine, National Cheng Kung University, Tainan, 701401 Taiwan; 5 Department of Health and Nutrition, Chia Nan University of Pharmacy and Science, No. 60, Sec. 1, Erren Rd., Rende Dist., Tainan City, 717301 Taiwan; 6 Department of Biochemistry and Molecular Biology, College of Medicine, National Cheng Kung University, Tainan, 701401 Taiwan; 7 National Institute of Cancer Research, National Health Research Institute, No. 367, Sheng-Li Rd, Tainan City, 704016 Taiwan; 8 Doctoral Program in Tissue Engineering and Regenerative Medicine, Biotechnology Center, National Chung Hsing University, Taichung, 402 Taiwan; 9 Center for Cancer Research, Kaohsiung Medical University, Kaohsiung, 807 Taiwan

**Keywords:** Breast cancer, STK11, CXCL1, Myeloid-derived suppressive cells

## Abstract

The tumor microenvironment in breast cancer is typically immunosuppressive. Combined neoadjuvant chemotherapy and immunotherapy is the standard of care for high-risk, triple-negative breast cancer patients, but it is not routinely applied to other breast cancer subtypes. Somatic mutation of STK11 (serine/threonine kinase 11), a tumor suppressor, occurs in the MSK-IMPACT platform in 1.9% of 3116 breast cases. A potential link between inactivation of STK11 and immune response regulation has been suggested in breast cancer, but the impact of STK11 suppression on tumor–immune interactions remains under investigation. In this study, we established *Stk11*-knockout (*Stk11*-KO) mouse breast cancer cell lines, performed RNA sequencing and cytokine array analysis to assess alterations in gene expression and cytokine profiles. Our results revealed significant enrichment of several immune-related pathways and a marked increase in C-X-C motif chemokine ligand 1 (Cxcl1) expression in response to *Stk11* knockout. Moreover, analysis of breast cancer patient samples showed an inverse association between the plasma CXCL1 levels and STK11 expression. To evaluate the in vivo effects of STK11 loss, we established an orthotopic breast cancer model in immunocompetent female mice. Tumors derived from *Stk11*-KO mouse breast cancer cells demonstrated greater tumorigenicity. In addition, mice-bearing *Stk11*-KO tumors exhibited elevated levels of circulating polymorphonuclear myeloid-derived suppressive cells. In summary, STK11 suppression enhances immune-related pathways and promotes Cxcl1 expression, correlating with the expansion of immunosuppressive MDSC populations. Our findings suggest that targeting STK11-associated immunosuppressive mechanisms may provide a novel therapeutic option for *STK11*-deficient breast cancer patients.

**Supplementary Information:**

The online version contains supplementary material available at 10.1007/s00262-025-04189-8.

## Introduction

The age-standardized incidence rate of breast cancer has increased year by year. In 2021, the point prevalence of female breast cancer was 450.64 (95% CI 427.02–475.96) per 100,000 population [1]. De-escalation of surgery from modified radical mastectomy to breast-conserving surgery with sentinel lymph node biopsy is the trend of treatment [2, 3] with the application of new local therapy to replace the operation, for example, percutaneous microwave ablation [4]. The trend of systemic therapy included a substantial rise in chemotherapy utilization and the introduction of new therapeutic agents [2]. Genetic factors (microRNAs or long non-coding RNAs) and epithelial–mesenchymal transition (EMT) regulators are responsible for cancer progression and have become potential treatment targets for breast cancer patients [5–9]. Trophoblast cell surface antigen 2 (Trop-2) is overexpressed in breast cancer, and antibody–drug conjugates against Trop-2, Sacituzumab govitecan, are approved by the US Food and Drug Administration (FDA) for patients with pre-treated metastatic breast cancer [10]. The FDA has approved several immune checkpoint inhibitors (ICIs) for cancer treatment. Notably, the KEYNOTE-522 trial demonstrated that combining pembrolizumab (an anti-programmed death-1 (PD-1) monoclonal antibody) with chemotherapy is effective as neoadjuvant therapy for high-risk, triple-negative breast cancer (TNBC) [11] and programmed death-ligand 1 (PD-L1)-positive metastatic TNBC [12]. Hormone receptor-positive breast cancer is typically characterized as an immunologically "cold" tumor due to low levels of tumor-infiltrating immune cells and an immunosuppressive tumor microenvironment (TME) [13]. Reprogramming the TME to convert "cold" tumors into "hot" ones has been shown to improve the efficacy of immunotherapies in patients with metastatic breast cancer refractory to conventional treatments [14]. Moreover, identifying predictive biomarkers is critical for selecting the appropriate patients for ICI therapy. While PD-L1/PD-1, microsatellite instability (MSI), tumor mutational burden (TMB), and aminoacyl tRNA synthetase complex interacting with multifunctional protein 2 (AIMP2) are well-established biomarkers, their clinical application remains limited [15, 16], highlighting the urgent need to identify alternative markers.

The *STK11* (serine/threonine kinase 11) gene encodes liver kinase B1 (LKB1), a key regulator of AMP-activated protein kinase (AMPK)/mammalian target of rapamycin complex 1 (mTORC1) signaling pathway. *STK11* is a tumor suppressor, and its loss-of-function mutations are implicated in Peutz–Jeghers syndrome (PJS) and various human cancers [17]. Across pan-cancer analysis, *STK11* alterations occur in approximately 1.35% of cases [18] and 3.5% the MSK-IMPACT Clinical Sequencing Cohort (www.cbioportal.org). In non-small-cell lung cancer (NSCLC), pathogenic *STK11* mutations are associated with poor prognosis in the *KRAS*-mutated subtype and have emerged as predictive biomarkers for primary resistance to ICIs [19, 20]. In fact, *STK11* mutations are among the most frequent drivers of ICI resistance in *KRAS*-mutant lung adenocarcinoma [21]. In breast cancer, *STK11* is considered a syndromic gene, and pathogenic germline variants of *STK11* are observed in 0.0087% of unselected breast cancer patients [22]. Somatic mutation of *STK11* occurs in 1.9% of 3116 breast cases in the MSK-IMPACT® database (Memorial Sloan Kettering Cancer Center-Integrated Mutation Profiling of Actionable Cancer Targets) (www.cbioportal.org). A small cohort study of the PJS families in Indian showed that all four breast cancer patients are estrogen receptor (ER)-positive and human epidermal growth factor receptor 2 (HER2)-negative [23]. However, there is no previous report about the correlation between breast cancer subtype and sporadic somatic mutation of *STK11*. Genomic inactivation of *STK11* has been linked to ICI resistance in metastatic breast cancer patients with all subtypes [24], and *STK11* mutations have been detected in circulating tumor cells from all subtypes of breast cancer patients [25]. Several clinical trials are currently conducting ICI therapies in *STK11*-mutated breast cancer, including all subtypes of patients [26].

Downregulation of STK11 promotes tumorigenesis across various cancers. In lung tumors, STK11 deficiency enhances neutrophil recruitment and pro-inflammatory cytokine expression in the TME, leading to T-cell suppression [27]. The immunosuppressive TME is also enriched in tumor-associated macrophages (TAMs) and myeloid-derived suppressive cells (MDSCs). Elevated MDSC accumulation has been observed in *STK11*-mutated PJS polyps compared to sporadic polyps, implicating an immunobiological link to cancer susceptibility following *STK11* loss [28]. Based on these findings, we hypothesized that downregulation of *STK11* in breast cancer cells activates immune-related pathways and fosters an immunosuppressive microenvironment, thereby promoting tumor progression.

## Methods

### Breast cell lines

The M158, NF639, and PY8119 mouse cancer cells were obtained from ATCC®. Cells were maintained in DMEM containing 10% fetal bovine serum, glutamine, and antibiotics. Single-guide RNAs (sgRNAs) were obtained from the RNAi core (National RNAi Core Facility, Academia Sinica, Taipei, Taiwan). The sgRNAs targeting the exon 1 (*Stk11*-sgRNA1: GCA CCG CAT CGA CTC CAC CG) and exon 3 (*Stk11*-sgRNA2: GTG ATG GAG TAC TGC GTA TG) in the coding DNA sequence of the mouse S*tk*11 were constructed into a lentiviral vector. Plasmids were transfected into the cancer cells using Lipofectamine. After transfection, cells were selected with puromycin (5 μg/mL) for 8 weeks to establish stable *Stk11*-knockout (KO) clones. The *Stk11-*KO was confirmed by Western blotting.

### Cytokine array

Cell lysates were prepared from parental breast cancer cell lines (M158, NF639, and PY8119) and their corresponding *Stk11*-KO derivatives. A Mouse Cytokine Antibody Array Kit C3 (RayBiotech, Norcross, GA) was used according to the manufacturer’s instructions. The protein sample was applied to the pre-coated antibody array membranes and incubated at room temperature for 4 h. After washing, a biotinylated antibody cocktail was added to each well and incubated for 2 h. Subsequently, HRP-conjugated streptavidin was applied, and the signal was detected using a chemiluminescent blot imaging system.

### Enzyme-linked immunosorbent assay (ELISA)

Plasma samples were obtained from the Human Biobank, Research Center of Clinical Medicine, National Cheng Kung University Hospital, with appropriate written informed consent, under protocols approved by the Institutional Review Board of National Cheng Kung University Hospital (IRB number: A-ER-109-501). Anonymous plasma samples from healthy volunteers were also used. A total of 122 breast cancer patients and 22 healthy volunteers were collected from January 1, 2010, to December 31, 2019. The study adhered to the Declaration of Helsinki. Enzyme-linked immunosorbent assay (ELISA) was performed using a commercial kit (R&D, Minneapolis, MN) per the manufacturer’s instructions. Standard recombinant human C-X-C motif chemokine ligand 1 (hCXCL1) or appropriately diluted plasma samples were used. A peroxidase substrate was added after incubation with a peroxidase-conjugated secondary antibody for 1 h at room temperature. After a final 30 min incubation, the colorimetric reaction was stopped by the addition of 50 μL stop solution, and absorbance was measured at 450 nm using a microplate reader.

### RNA sequencing

Total RNA was extracted from parental breast cancer cell lines (M158, NF639, and PY8119) and their corresponding *Stk11*-KO derivatives using Trizol® Reagent (Invitrogen, Waltham, MA), following the manufacturer's instructions. The RNA was quantified and qualified. mRNA libraries were prepared using the SureSelect XT HS2 mRNA Library Preparation kit (Agilent, Santa Clara, CA), and size selection was performed using AMPure XP beads (Beckman Coulter, Pasadena, CA). Sequencing was conducted using Illumina's sequencing-by-synthesis technology (Illumina, San Diego, CA). FASTQ sequencing reads were generated using Welgene Biotech's pipeline, based on Illumina's base-calling software bcl2fastq v2.20. Differential expression analysis was performed to compare *Stk11*-KO and parental controls. Genes with low expression level (< 0.3 transcripts per million) in either or both samples were excluded from the analysis. *P*-values were calculated using the DESeq package in R, and the Benjamini–Hochberg procedure was applied to determine the *P*-value distribution and to estimate the *q*-value (false discovery rate, FDR). Differentially expressed genes were identified using a *P*-value threshold of 0.05 and an absolute Log_2_ (fold change) cutoff of 1.00.

### Bioinformatics

RNA sequencing data from 1217 breast cancer samples were obtained from the Illumina platform, and raw data were downloaded from The Cancer Genome Atlas Breast Invasive Carcinoma in Genomic Data Commons Data Portal (GDC TCGA-BRCA). The results were reanalyzed using the latest Human Genome Assembly hg38 and reorganized by the University of California, Santa Cruz Xena team (https://xena.ucsc.edu). We selected the upper quartile normalized fragments per kilobase of transcript per million mapped reads (HTSeq-FPKM-UQ). Expression data for selected target genes were extracted, and corresponding patient survival information was retrieved. The low or high STK11 levels were divided by the median. To explore the protein–protein interaction (PPI) network associated with the target genes, we used STRING v12.0 (https://string-db.org). The *k*-means clustering algorithm was applied to classify proteins into functionally distinct groups based on their interaction patterns.

### Gene set enrichment analysis (GSEA)

We performed GSEA to identify enriched pathways associated with gene activity in parental breast cancer cell lines and their *Stk11*-KO counterparts, using transcriptomic data obtained from RNA sequencing. The analysis was conducted using GSEA 4.1.0 (https://www.gsea-msigdb.org). For each gene set, a normalized enrichment score (NES) and FDR* q*-value were calculated. A *q*-value < 0.25 was used as the boundary criterion for significance. Additionally, nominal *P*-value < 0.05 or NES > 1 was applied as a threshold for identifying significantly enriched gene sets.

### Orthotopic mouse model of breast cancer

For all animal experiments, 12 week-old immunocompetent C57BL/6 mice (body weight 18.7–21.0 g) were purchased from the Laboratory Animal Center, College of Medicine, National Cheng Kung University (Tainan, Taiwan), and maintained under pathogen-free conditions. The animal study was approved by the Laboratory Animal Center, College of Medicine, National Cheng Kung University (NCKU-110058), and performed from Aug 30, 2022, until Oct 14, 2022. Mice were anesthetized by intraperitoneal injection of Zoletil (Virbac, Carros, France) and Rompun (Bayer, Leverkusen, Germany). A small incision was made in the right lower abdomen to expose the 4th mammary gland. A total of 2.5 × 10^5^ PY8119 parental cells or their *Stk11*-KO counterparts, suspended in 50-μL serum-free medium, were injected into the mammary gland (n = 6 in each group). The incision was closed, and the mice were monitored regularly. Tumor growth was measured thrice weekly using two perpendicular diameters. Tumor volume was calculated as length × width^2^/2. On day 20, blood samples were collected via submandibular plexus puncture, and peripheral blood mononuclear cells were isolated for flow cytometry analysis. According to the regulatory guidelines of the Laboratory Animal Center at our institution, mouse models must be euthanized if the tumor diameter exceeds 20 mm. Mice were sacrificed on day 22 because the largest tumor in the *Stk11*-KO group measured 19 mm on day 21. Euthanasia of mice was performed by carbon dioxide overdose. Tumor samples were collected to measure tumor weight and further analyzed tumor-infiltrating immune cells. The animal study adhered to the ARRIVE guidelines. We selected a small sample size, and all mice were handled carefully without any unexpected sacrifices. The mice were randomized for injection of PY8119 parental cells or their *Stk11*-KO counterparts using the standard = RAND() function in Microsoft Excel.

### Flow cytometry analysis

Circulating and tumor-infiltrating immune cells were isolated and incubated overnight at 4 °C with commercially available antibodies: anti-CD3-FITC (Fluorescein Isothiocyanate) (clone 145-2C11), anti-CD4-PE (Phycoerythrin) (clone H129.19), anti-CD8-PE (clone 53–6.7), anti-CD107a-Cy7 (Cyanine 7) (clone 1D4B), anti-CD11b-PE (clone M1/70), anti-Ly6C-BV421 (Brilliant Violet 421) (clone AL-21), and anti-Ly6G-FITC (clone 1A8) (BD Biosciences, Franklin Lakes, NJ, USA). After staining, 1 mL of staining buffer was added, and the cells were analyzed by flow cytometry (BD Biosciences). CD3^+^CD4^+^ cells were classified as regulatory T cells (Tregs), and CD3^+^CD8^+^ cells were identified as cytotoxic T lymphocytes (CTLs). Myeloid-derived suppressor cells (MDSCs) were further subdivided into polymorphonuclear MDSCs (PMN-MDSCs), defined as CD11b^+^Ly6C^−^Ly6G^+^, and monocytic MDSCs (M-MDSCs), defined as CD11b^+^Ly6C^+^Ly6G^−^. Flow cytometry data were analyzed using CytExpert, version 2.4 (Beckman).

### Statistics

All statistical analyses were performed using STATA version 16.1 (StataCorp, College Station, TX, USA). Univariate comparisons between categorical variables were performed using the Chi-square test. The nonparametric Kruskal–Wallis test was used for continuous variables that did not follow a normal distribution. Pairwise correlation coefficients between two variables were calculated using Pearson’s method. The Wilcoxon rank-sum test compared the difference in cytokine array between *Stk11*-KO and parental cells. Survival curves were generated using the Kaplan–Meier method and compared between groups using the log-rank test. A median split was applied for the Kaplan–Meier analysis to convert the continuous variable into binary variables for group comparison. According to our previous study, the mean tumor size was 600 mm^3^ in the orthotopic mouse model from PY8119 parental breast cancer cells with a standard deviation of about 250 mm^3^. We planned a study with PY8119 parental and *Stk11*-KO cells, with one control per experimental subject. We will need to study six experimental and six control subjects to reject the null hypothesis that the population means of the experimental and control groups are equal with probability (power) 0.95. The Type I error probability associated with this test of the null hypothesis is 0.05. The calculation was performed by the PS program developed by the Department of Biostatistics, Vanderbilt University, version a57e8c3 (https://vbiostatps.app.vumc.org/). For multiple testing in Fig. 6, the Benjamini–Hochberg procedure adjusted the P value [29]. A *P*-value < 0.05 was considered statistically significant.

## Results

### Alteration of downstream genes after *Stk11* knockout

**Fig. 1:**
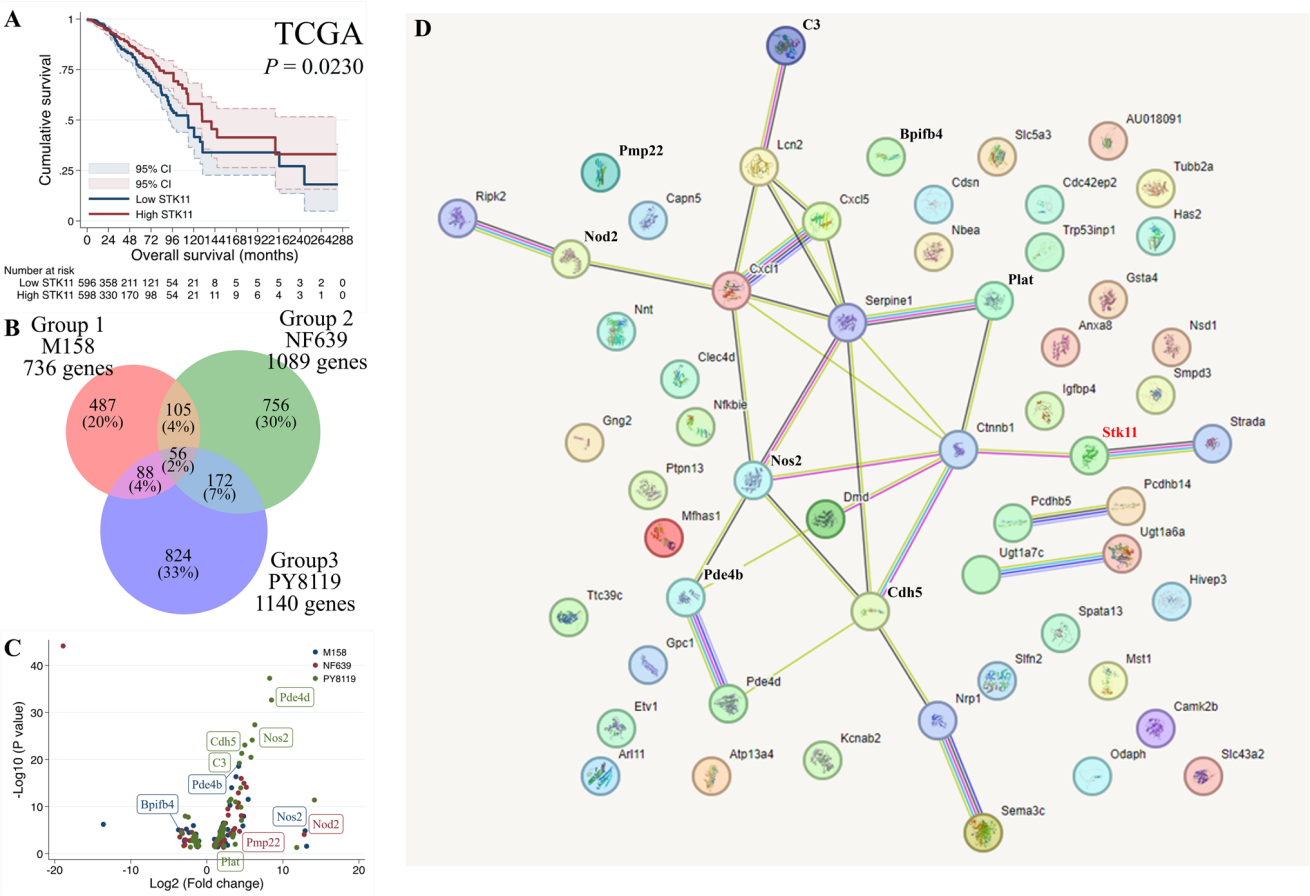
Consequence of low *STK11* in breast cancer. **A** Overall survival curve according to the mRNA of *STK11* from The Cancer Genome Atlas Breast Invasive Carcinoma in Genomic Data Commons Data Portal (GDC TCGA-BRCA) dataset. The patients with high *STK11* expression have a better survival than those with low expression (*P* = 0.0230). **B**-**D** Differential expression genes (DEGs) of *Stk11*-KO cells compared with their parental counterparts based on RNA sequencing analysis. **B** Venn diagram showing DEGs identified in *Stk11*-KO versus parental cells for the three mouse breast cancer cell lines, Group 1: M158, Group 2: NF639, and Group 3: PY8119. A total of 56 DEGs were shared among all three groups. **C** Volcano plot illustrating the expression patterns of the 56 DEGs. DEGs in M158 cells are shown in blue, NF639 in red, and PY8119 in green. **D** Predicated protein–protein interaction (PPI) network of *Stk11* and the 56 DEGs

RNA sequencing data from 1217 breast cancer samples were retrieved from the GDC TCGA-BRCA dataset. The patients with low expression of *STK11* mRNA had a worse overall survival (*P* value = 0.023, Fig. 1A). The results confirmed the clinical significance of low *STK11* levels in breast cancer patients. Therefore, we used a cell line model for further investigation.

Knockout of the S*tk11* gene was performed by sgRNA transfection in three mouse breast cancer cell lines: M158, NF639, and PY8119. RNA sequencing was performed on *Stk11*-KO cells and their parental counterparts, and differential expression genes (DEGs) were identified. A total of 736 DEGs were detected in M158, 1089 in NF639, and 1140 in PY8119 cells (Fig. 1A). A Venn diagram revealed 56 overlapping genes shared across all three cell lines (Fig. 1A and Supplementary Table 1). The expression patterns of these 56 common genes are illustrated in volcano plots for each cell line (Fig. 1B). Protein–protein interaction (PPI) analysis was performed using the STRING v12.0 database to explore functional interactions. *Stk11* was found to be functionally linked to *Ctnnb1* (encoding β-catenin), with further connections extending to additional proteins (Fig. 1C). Among these DEGs, seven genes (*Nod2*, *Nos2*, *C3*, *Cdh5*, *Plat*, *Pmp22*, and *Pde4b*) were consistently upregulated across all three cell lines. Conversely, only *Bpifb4* was downregulated in three cell lines (Supplementary Table 2).

**Fig. 2:**
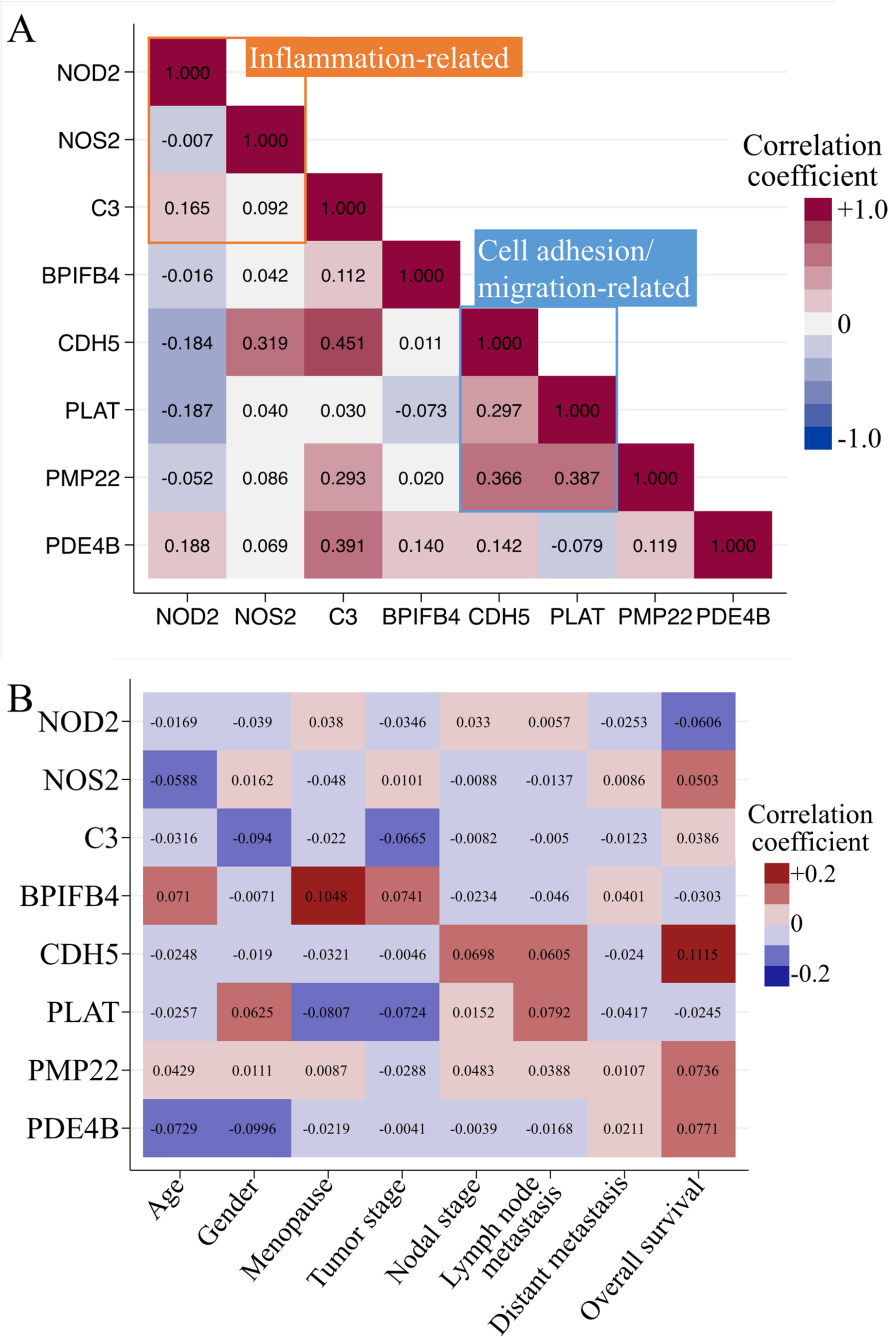
Correlation analysis of target gene expression in breast cancer. **A** Correlation matrix illustrating pairwise correlation coefficients among selected genes based on RNA sequencing data. Inflammation-related genes are indicated with an orange box, and cell adhesion and migration-related genes are indicated with a blue box, showing functional grouping. **B** Correlation heatmap drawing the correlation between target gene expression and clinical or demographic characteristics of breast cancer patients. The value of the correlation coefficient is shown in the center of each cell. Red indicates a positive correlation, and blue indicates a negative correlation

RNA sequencing data from the TCGA-BRCA dataset were analyzed. In *Stk11-*KO cells, cell adhesion and migration-related genes were consistently upregulated. In contrast, the expression patterns of inflammation-related genes varied (Fig. 2A). Clinical and demographic data of breast cancer patients were analyzed. Correlation between gene expression and patient characteristic factors was weak. However, high expression of *CDH5* was positively correlated with poor overall survival, whereas *NOD2* expression showed a negative correlation with survival (Fig. 2B).

### Enhancement of immune-related pathways after *Stk11* knockout

**Fig. 3:**
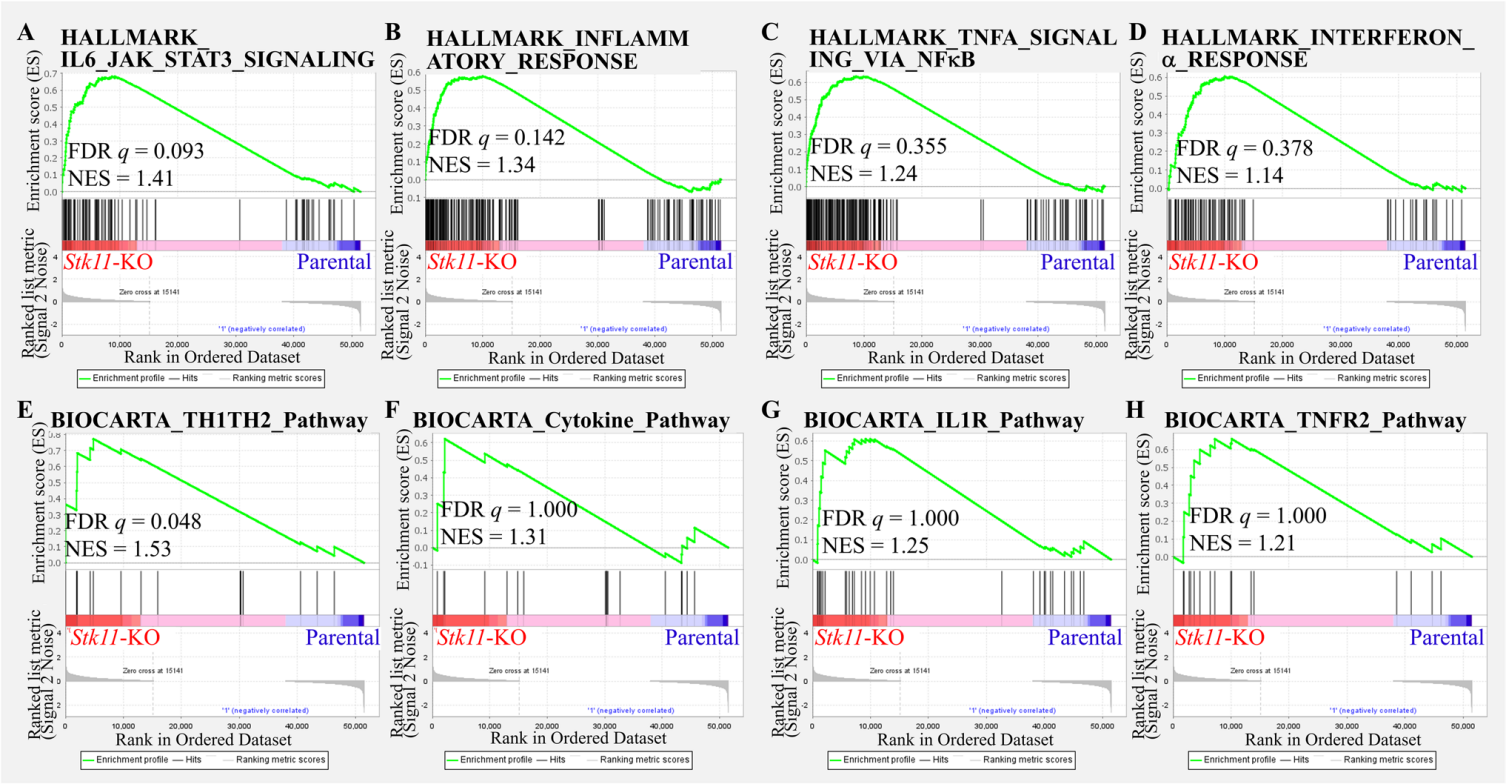
Gene set enrichment analysis (GSEA) of immune-related pathways in *Stk11*-KO cells versus their parental counterparts. GSEA was performed using HALLMARK and BIOCARTA gene sets on RNA sequencing data from three mouse breast cancer cell lines (PY8119, M158, and NF639). The analysis compared *Stk11*-KO cells with their respective parental counterparts to identify enrichment of immune-related pathways. Representative enrichment plots are shown for the following pathways: **A** IL6_JAK_STAT3_SIGNALING. **B** INFLAMMATORY_RESPONSE. **C** TNFA_SIGNALING_VIA_NFκB. **D** INTERFERON_ALPHA_RESPONSE. **E** TH1_TH2 _SIGNALING. **F** INFLAMMATORY_RESPONSE. **G** IL1R_PATHWAY. **H** TNFR2_PATHWAY

**Fig. 4:**
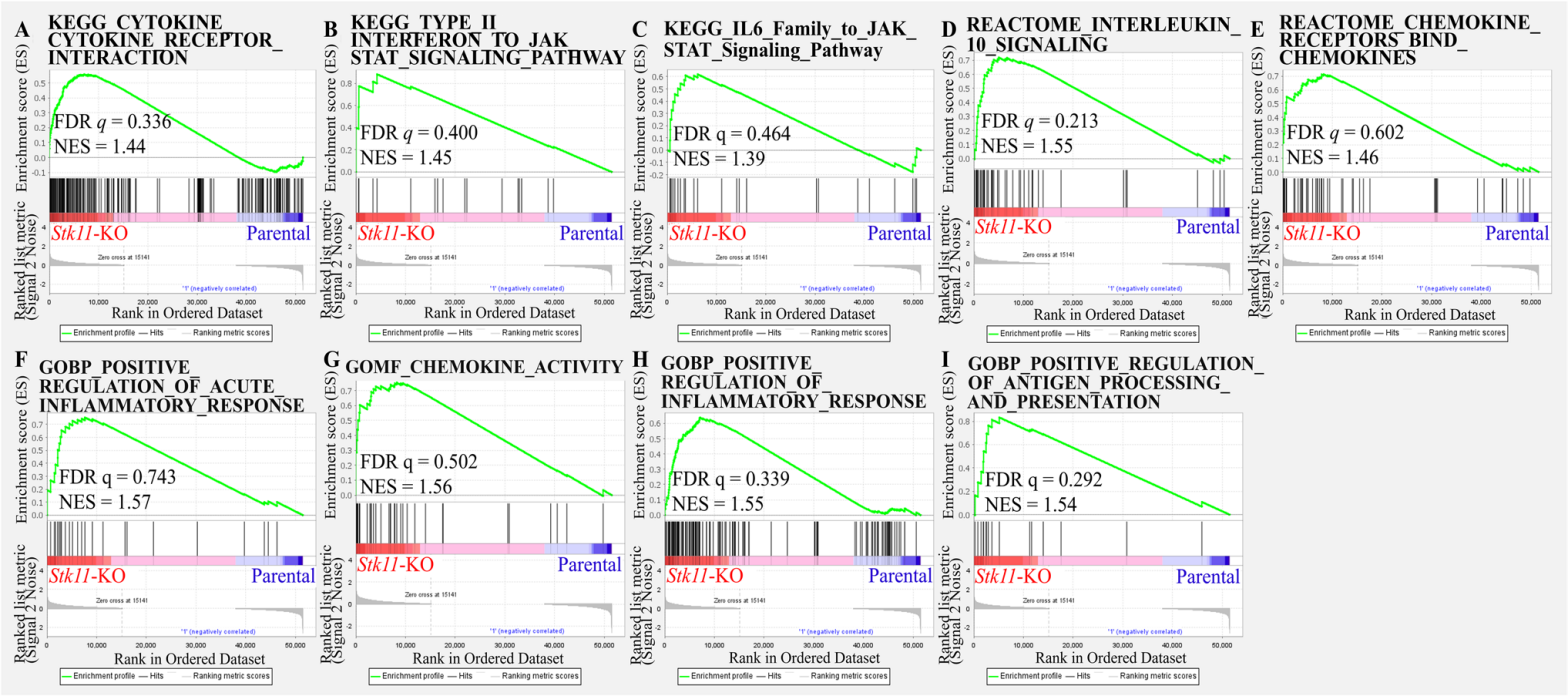
Gene set enrichment analysis (GSEA) of immune-related pathways in Stk11-KO cells versus their parental counterparts using Kyoto Encyclopedia of Genes and Genomes (KEGG), REACTOME, and GENE Ontology (GO) gene sets. GESA was performed on RNA sequencing data from *Stk11*-KO cells and their parental counterparts derived from three mouse breast cancer cell lines (PY8119, M158, and NF639). Enrichment analysis included gene sets from the KEGG, REACTOME, and GO databases. Representative enrichment plots are shown for the following pathways and biological functions: **A** CYTOKINE_CYTOKINE_RECEPTOR_INTERACTION. **B** TYPE_II_INTERFERON_TO_JAK_STAT_SIGNALING_PATHWAY. **C** IL6_FAMILY_TO_JAK_STAT_SIGNALING-PATHWAY. **D** INTERLEUKIN_10_ SIGNALING. (**E**) CHEMOKINE_RECEPTORS_BIND_CHEMOKINES. **F** Gene ontology biological process (GOBP)_REGULATION_OF_ACUTE_ INFLAMMATORY_RESPONSE. **G** Gene ontology molecular function (GOMF)_CHEMOKINE_ACTIVITY. **H** GOBP_POSITIVE_REGULATION_OF_ INFLAMMATORY_RESPONSE. **I** GOBP_POSITIVE_REGULATION_OF_ ANTIGEN_PROCESSING_AND_PRESENTATION

**Fig. 5:**
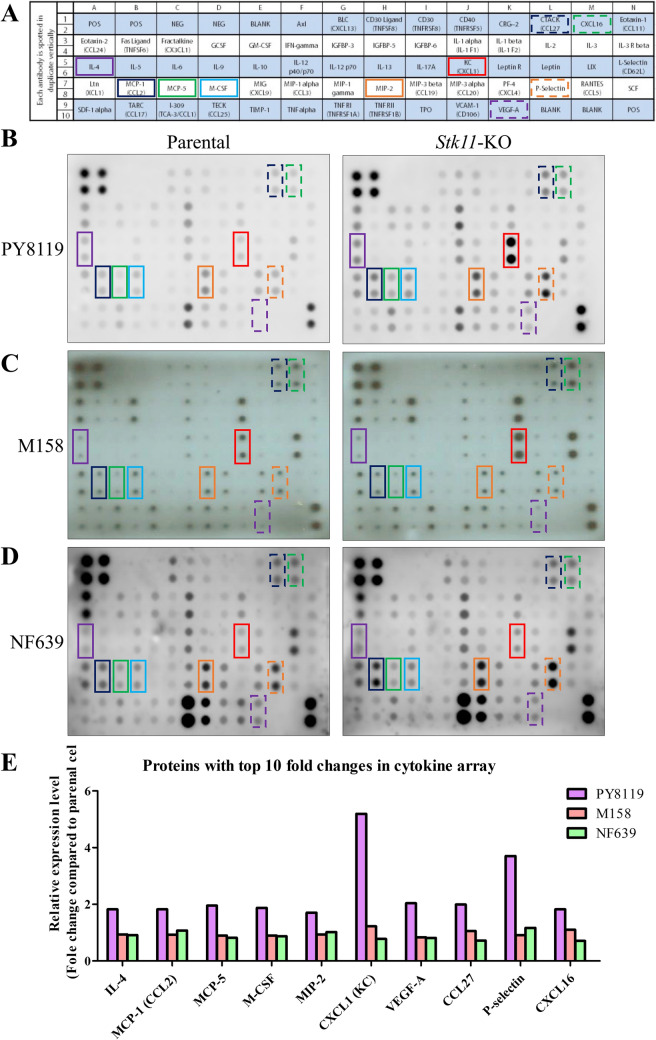
Cytokine array analysis of immune-related protein expression in *Stk11*-KO mouse breast cancer cell lines and their parental counterparts. **A** Array map. **B**-**D** Representative cytokine array blots for PY8119 **B**, M158 **C**, and NF639 **D** cell lines, comparing *Stk11*-KO cells with their parental counterparts. **E** Semiquantitative analysis of protein expression with a top tenfold change. Signal intensities were measured and expressed as fold changes relative to parental cells, based on the dot intensity from the cytokine array. The Wilcoxon rank-sum test compared the difference between *Stk11*-KO and parental cells. Abbreviations: CTACK/CCL27, cutaneous T cell-attracting chemokine/C–C motif chemokine ligand 27; CXCL1/KC, chemokine (C-X-C motif) ligand 1; CXCL16, chemokine (C-X-C motif) ligand 16; IL-4, interleukin-4; M-CSF, macrophage colony-stimulating factor; MCP-2/CCL2, monocyte chemoattractant protein-1/C–C motif chemokine ligand 2; MCP-5, monocyte chemoattractant protein-5; MIP-2, macrophage inflammatory protein-2; and VEGF-A, vascular endothelial growth factor-A

Immune-related pathways were significantly enriched in *Stk11*-KO cells, as identified using Gene Set Enrichment Analysis (GSEA) (Figs. 3 and 4). To validate these transcriptomic findings, we used cytokine antibody arrays to examine the expression of immune-related proteins in these mouse breast cancer cell lines (Fig. 5A). Three mouse cell lines were used (PY8119 in Fig. 5B, M158 in Fig. 5C, and NF639 in Fig. 5D). Semiquantitative comparison between *Stk11*-KO and parental cells was calculated. The proteins with top tenfold changes in *Stk11*-KO than parental cells were marked, including interleukin-4 (IL-4), monocyte chemoattractant protein-1 (MCP-1/CCL2), monocyte chemoattractant protein-5 (MCP-5), macrophage colony-stimulating factor (M-CSF), macrophage inflammatory protein-2 (MIP-2), CXCL1 (also named as keratinocyte-derived cytokine, KC), vascular endothelial growth factor-A (VEGF-A), cutaneous T cell-attracting chemokine (CTACK/CCL27), P-selectin, and CXCL16. Notably, Cxcl1 expression was consistently increased across all three cell lines (Fig. 5E), implying a potential role of Cxcl1 as a common downstream effector in response to *Stk11* loss.

To evaluate the clinical relevance of CXCL1, we determined its plasma levels in healthy controls (n = 22) and breast cancer patients (n = 122) using ELISA (Supplementary Fig. 1) We found that CXCL1 levels were similar between healthy individuals and patients (Supplementary Fig. 1A), and showed no correlation with age or tumor size (Supplementary Fig. 1B and C). Interestingly, patients with grade I tumors exhibited significantly higher plasma CXCL levels (Supplementary Fig. 1D, *P* value = 0.038). At the same time, no association was found with other pathological features (Supplementary Fig. 1E–G) or overall survival (Supplementary Fig. 1H). Moreover, an inverse correlation between STK11 and CXCL1 expression was observed. Patients with high STK11 expression in immunohistochemical (IHC) analysis had lower plasma CXCL1 levels (median: 45.049 pg/mL; range: 26.015–304.805 pg/mL), compared to those with low STK11 expression (median: 60.434 pg/mL; range: 28.325–177.551 pg/mL) (Supplementary Fig. 1I). The reciprocal relationship between STK11 and CXCL1 is consistent with the results of cytokine array, further supporting a regulatory link.

### *Stk11* suppression alters populations of tumor-infiltrating and circulating immune cells

**Fig. 6:**
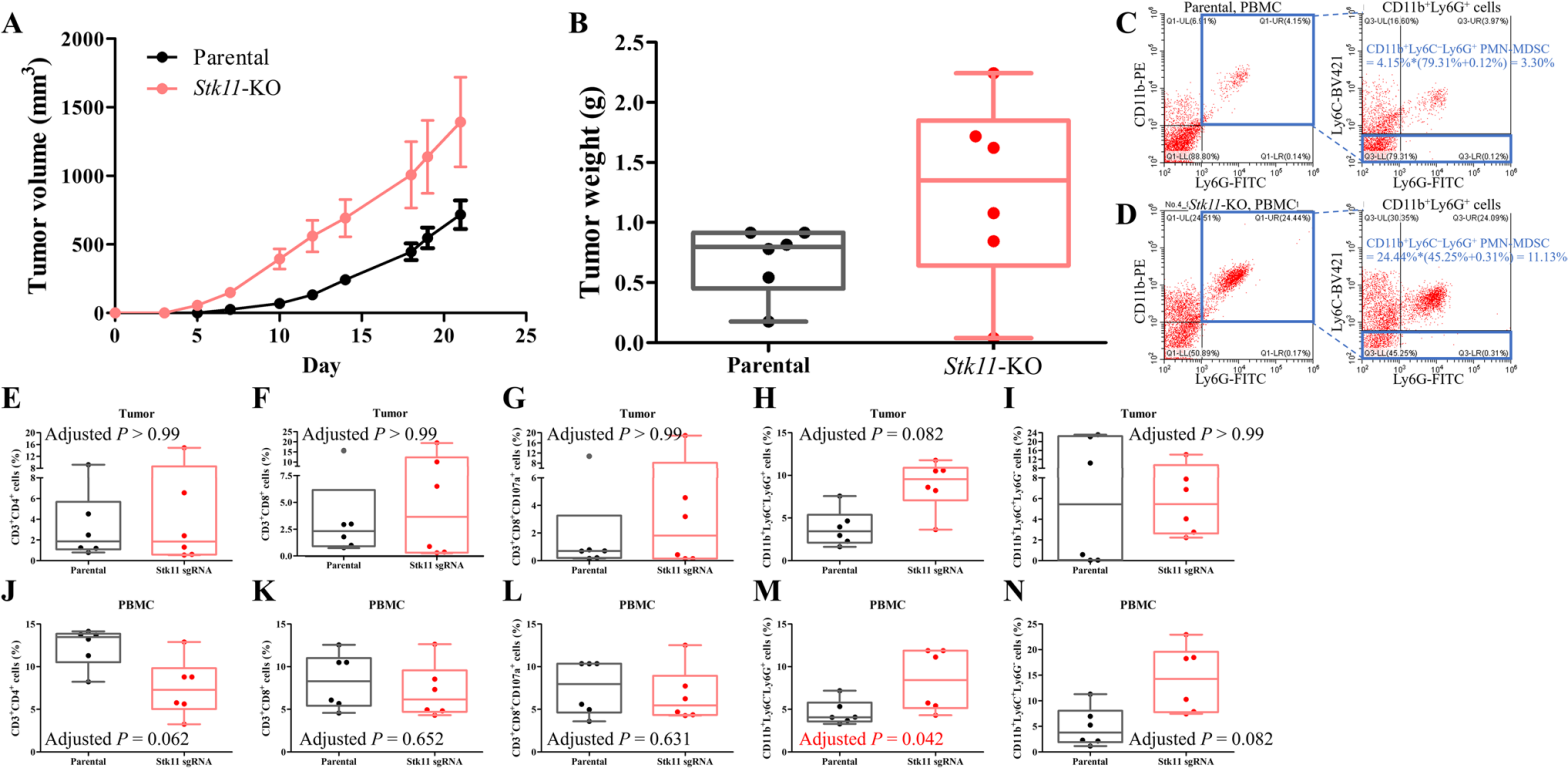
Immune profiling in an orthotopic breast cancer mouse model using *Stk11*-KO cells and their parental counterparts derived from PY8119. Cells were orthotopically injected into the mammary gland of C57BL/6 mice (n = 6 per group). **A** Tumor volume was monitored throughout the experiment. **B** Tumor weight was assessed at day 22 after sacrifice. **C**–**N** Circulating and tumor-infiltrating immune cells are collected at day 22 after sacrifice and examined by flow cytometry analysis. **C** Examples of CD11b^+^Ly6C^−^Ly6G^+^ polymorphonuclear myeloid-derived suppressive cells (PMN-MDSCs) in peripheral mononuclear cells (PBMCs) from tumor-bearing mice of PY8119 parental cells. Left plot: Double staining with CD11b-PE and Ly6G-FITC in flow cytometry. The upper right quadrant showed CD11b^+^Ly6G^+^ cells. Right plot: Triple staining with CD11b-PE, Ly6C-BV421, and Ly6G-FITC in flow cytometry. CD11b^+^Ly6G^+^ cells from the upper right quadrant of the left plot are gated by Ly6C-BV421 staining. The proportion of CD11b^+^Ly6C^−^Ly6G^+^ PMN-MDSCs is the percentage of the upper right quadrant of the left plot multiplied by the rate of Ly6C^−^ cells (lower left and lower right quadrants of the right plot). **D** Examples of circulating CD11b^+^Ly6C^−^Ly6G^+^ PMN-MDSCs from tumor-bearing mice of *Stk11*-KO cells. **E**-**I** Tumor-infiltrating immune cells were examined by flow cytometry. **C** CD3^+^CD4^+^ lymphocytes. **D** CD3^+^CD8^+^ cytotoxic T cells. **E** CD3^+^CD8^+^CD107^+^ activated cytotoxic T cells. **F** CD11b^+^Ly6C^−^Ly6G^+^ PMN-MDSCs. **G** CD11b^+^Ly6C^+^Ly6G^−^ monocytic myeloid-derived suppressive cells (M-MDSCs). **J**-**N** Blood samples were collected on day 20. Circulating immune cells in peripheral blood were examined by flow cytometry: **H** CD3^+^CD4^+^ lymphocytes. **I** CD3^+^CD8^+^ cytotoxic T cells. **J** CD3^+^CD8^+^CD107^+^ activated cytotoxic T cells. **K** CD11b^+^Ly6C^−^Ly6G^+^ PMN-MDSCs. **L** CD11b^+^Ly6C^+^Ly6G^−^ M-MDSCs. Abbreviations: M-MDSCs, monocytic myeloid-derived suppressive cells; PBMCs, peripheral mononuclear cells; and PMN-MDSCs, polymorphonuclear myeloid-derived suppressive cells

CXCL1, a chemokine well documented for its role in regulating tumor-infiltrating immune cells [30], was found to be upregulated after suppression of STK11. GSEA further confirmed the enrichment of immune-related pathways in *Stk11*-KO cells, suggesting the hypothesis that STK11 modulates tumor–immune interactions. To investigate this regulatory role in vivo, we employed an orthotopic breast cancer model using immunocompetent C57BL/6 mice. PY8119 parental and *Stk11*-KO cells were injected into the mammary fat pads, and *Stk11*-KO tumors developed significantly larger tumor volumes than those derived from parental cells (Fig.6A and B).

On day 20, blood samples were collected, and peripheral blood mononuclear cells (PBMCs) were isolated for immune profiling. Examples of flow cytometry are shown in Fig. 6C and D. Tumor-infiltrating (Fig. 6E-I) and systemic circulating (Fig. 6J-N) immune cells were analyzed using flow cytometry. The following cell populations were identified as CD3^+^CD4^+^ lymphocytes, CD3^+^CD8^+^ cytotoxic T cells, CD3^+^CD8^+^CD107^+^ activated cytotoxic T cells, CD11b^+^Ly6C^−^Ly6G^+^ PMN-MDSCs, and CD11b^+^Ly6C^+^Ly6G^−^ M-MDSCs. Only CD11b^+^Ly6C^−^Ly6G^+^ PMN-MDSCs among tumor-infiltrating immune cells were increased in *Stk11*-KO tumors with an adjusted *P* value = 0.082 (Fig. 6H). In the peripheral blood, circulating CD3^+^CD4^+^ lymphocytes were slightly decreased in mice-bearing *Stk11*-KO tumors (Fig. 6J), while PMN-MDSCs (CD11b^+^Ly6C^−^Ly6G^+^) were elevated significantly with an adjusted *P* value = 0.042 (Fig. 6M). These findings indicate that *STK11* suppression enhanced systemic and intertumoral immunosuppressive myeloid cell populations, contributing to accelerated tumor progression.

## Discussion

*STK11* is a well-documented tumor suppressor gene, and its mutation results in loss of function. Although the incidence of *STK11* mutation is relatively low in breast cancer patients, it has been associated with an increased odds ratio for poor overall survival. In the present study, we investigate downstream effectors of STK11 suppression using RNA sequencing and cytokine array analysis in murine breast cancer cell lines. Our results revealed that STK11 knockout led to the upregulation of immune-related genes and pathways, and consistently increased the expression of CXCL1 across all three cell lines. Moreover, we employed an orthotopic breast cancer model in immunocompetent mice to explore the immunological consequences of STK11 loss. Mice injected with *Stk11*-KO cells developed significantly larger tumors than those receiving parental cells. Flow cytometric analysis revealed that circulating CD11b^+^Ly6C^−^Ly6G^+^ PMN-MDSCs and CD11b^+^Ly6C^+^Ly6G^−^ M-MDSCs were increased dramatically in mice-bearing *Stk11*-KO tumors. Tumor-infiltrating CD11b^+^Ly6C^−^Ly6G^+^ PMN-MDSCs were also increased in the *Stk11*-KO group. These results suggest that STK11 loss promotes an immunosuppressive microenvironment, likely contributing to enhanced tumor growth and immune evasion.

Genomic inactivation of *STK11* has been identified as a predictive biomarker for ICI resistance in both metastatic breast cancer and NSCLC patients [24, 31]. This implies that the *STK11* mutant tumor may partially regulate the host immune system through modulating cytokine signaling. Several cytokines are known to be involved in tumor development and immune modulation, including tumor necrosis factor (TNF)-α, IL-6, IL-10, IL-12, IL-17, transforming growth factor (TGF)-β, and macrophage migration inhibitory factor (MIF) [32]. In lung cancer, suppression of inflammation genes has been reported in patients harboring *STK11* and *KRAS* co-mutations [33]. Dual inactivation of *STK11* and *KRAS* genes alters the tumor secretome, resulting in increased secretion of IL-6 [34]. In breast cancer, STK11/AMPK signaling has been shown to inhibit TGF-β transcription and downstream Smad phosphorylation, modulating TGF-β-mediated responses [35]. In endometrial cancer, STK11 inactivation enhances MCP-1/CCL2 expression, which increases immune cell recruitment [36]. In NSCLC, loss of STK11 leads to increased secretion of several C-X-C motif chemokines, reinforcing its role in immune regulation [37]. CXCL1 is a chemokine extensively documented for its role in modulating tumor-infiltrating immune cells [27]. CXCL1/CXCR2 signaling promotes the recruitment and accumulation of PMN-MDSCs in gastric cancer, leading to CD8⁺ T-cell exhaustion [38]. AMPK, a downstream effector of STK11, is activated by metformin. Metformin induces AMPK phosphorylation, suppressing CXCL1 secretion in esophageal squamous carcinoma cells and reducing MDSC migration [39]. In a previous study, treatment with Cxcl-1 neutralizing antibody abrogates the effect of colony-stimulating factor-1 receptor (CSF1R) inhibitor and dramatically reduces PMN-MDSC migration [40]. Consistent with these findings, our study demonstrated that *STK11* knockout in breast cancer cells led to the enrichment of immune-related pathways, spanning from cytokine-associated signaling to broader immune system regulatory networks. Furthermore, cytokine array analysis revealed significantly increased expression of Cxcl1 in *Stk11*-KO cells. The three cell lines used in the present study were derived from different mouse cancers: PY8119 from TNBC, M158 from c-MYC-overexpressed, and NF639 from c-neu-overexpressed mammary gland tumor. Our results demonstrate that suppression of STK11 enhances CXCL1 secretion and promotes PMN-MDSC recruitment, particularly in the murine TNBC PY8119 cell line, potentially via an AMPK-dependent pathway. In our results, plasma CXCL1 levels were similar between healthy individuals and patients. There was no correlation between plasma CXCL1 levels and age, tumor size, nodal stage, TNM stage, intrinsic subtypes, or recurrence-free survival of breast cancer patients. The patients with grade I tumors exhibited significantly higher plasma CXCL levels. The only significant finding was an inverse correlation between IHC STK11 staining and CXCL1 expression. Plasma CXCL1 levels fail to be a biomarker; however, our results confirmed that tumor-derived STK11 plays a pivotal role in regulating cytokine secretion, thereby contributing to the modulation of the tumor–immune microenvironment.

The tumor-infiltrating immune cells in breast cancer include tumor-infiltrating lymphocytes (TILs), TAMs, cancer-associated fibroblasts (CAFs), natural killer cells, dendritic cells, and MDSCs [41]. In our previous study, lower levels of CD3^+^CD8^+^ cytotoxic T cells were correlated with advanced cancer grade, extensive intraductal components, and positive lymphatic tumor emboli in breast cancer [42]. We also found the reciprocal relationship between CD3^+^CD8^+^ cytotoxic T cells and PMN-MDSCs in breast cancer patients. For example, the proportion of CD3^+^CD8^+^ cytotoxic T cells was higher in T1 than in T2 cancer, and the percentage of CD11b^+^CD14^−^/CD33^+^CD14^−^ PMN-MDSCs was lower in T1 than in T2 cancer [42]. S100A8/A9 heterodimer from PMN-MDSCs inhibits glycolysis and proliferation of CD8^+^ T cells, and PMN-MDSCs are upstream suppressive cells for CD8^+^ T cells exhaustion, inducing ICIs resistance of gastric cancer [38]. A significant increase in circulating MDSCs has been detected in cancer patients compared to healthy volunteers, and MDSC levels decrease after curative operation in breast cancer patients [43]. There are two major phenotypes of MDSCs, PMN-MDSCs and M-MDSCs. Tumor-infiltrating M-MDSCs have been shown to promote EMT of breast cancer cells, thereby facilitating tumor cell dissemination. In contrast, PMN-MDSCs residing in distant organs can reverse EMT, supporting metastatic tumor outgrowth [44]. MDSC accumulation has also been observed in premalignant polyps of patients with Peutz–Jeghers syndrome caused by germline STK11 deficiency [28]. Similarly, in *STK11*-deficient NSCLC mouse models, large numbers of PMN-MDSCs have been identified both within the locally tumor microenvironment and systemically in peripheral blood [37]. However, no study directly correlates STK11 expression in breast cancers with their relationships with tumor-infiltrating immune cells. In the present study, we found that CD11b^+^Ly6C^−^Ly6G^+^ PMN-MDSCs were significantly increased in the peripheral blood of mice-bearing *Stk11*-KO breast tumors, and slightly expanded locally within the tumor microenvironment. The findings are consistent with the previous reports in NSCLC and further support the promotional role of STK11 loss in immunosuppressive myeloid populations. Given their contribution to immune evasion, targeting PMN-MDSCs may represent a potential therapeutic strategy to defeat resistance to immune checkpoint inhibitors in *STK11*-deficient breast cancer.

Suppression of STK11 has been shown to promote cancer cell migration, invasion, and metastasis [45, 46]. In lung adenocarcinoma, STK11 depletion results in increased expression of mesenchymal markers and enhanced cell viability [47]. Knockdown of STK11 in cholangiocarcinoma cells activates Wnt/β-catenin signaling, thereby enhancing cell growth, migration, and invasion [48]. Mechanistically, STK11 plays a role in stabilizing focal adhesion by enhancing phosphorylation of focal adhesion kinase (FAK) [45]. STK11 farnesylation also induces actin stress fiber formation through the RhoA-ROCK pathway, thus regulating membrane dynamics [49]. Moreover, somatic missense mutation in *STK11* in human cancers impairs its tumor-suppressive function and promotes cell motility independently of its kinase activity [50]. *STK11* mutants have also been shown to downregulate genes involved in vesicle trafficking, cell adhesion, exocytosis, and cytokine production [50]. In our study, knockout of *Stk11* in murine breast cancer cell lines resulted in significant enrichment of cytoskeleton-related gene (*Bpifb4*), and cell adhesion/migration-related genes (*Cdh5*, *Plat*, and *Pmp22*), along with enhanced tumorigenicity in vivo. These results indicate that STK11 loss promotes a pro-metastatic phenotype through modulation of cytoskeletal and adhesion-related pathways. This suggests that restoring STK11 function or targeting its downstream effectors may offer a promising therapeutic approach for treating *STK11*-deficient cancers.

The present study used mouse breast cancer cell lines to investigate phenotypic alterations in response to *Stk11* knockout. We employed an orthotopic breast cancer model in immunocompetent mice to investigate the host immune responses. The models provide valuable mechanistic insights, but the differences between murine and human immune systems may limit our findings' clinical translation and applicability [51]. A small cohort of PJS families in India revealed that all four breast cancer patients were ER-positive and HER2-negative [23]. Somatic mutations in *STK11* have been identified as predictive biomarkers for ICI resistance in patients with metastatic TNBC [24]. However, no prior studies have reported a correlation between breast cancer subtypes and sporadic somatic mutations in *STK11*. To explore this relationship, we utilized three murine breast cancer models to assess cellular responses following *Stk11* suppression: PY8119 from TNBC, M158 from c-MYC-overexpressed, and NF639 from c-neu-overexpressed mammary gland tumor. M158 and NF639 cells fail to exhibit tumorigenicity in immunocompetent mice, limiting their utility for investigating tumor-infiltrating immune cells. In contrast, the PY8119 TNBC model demonstrated a more pronounced alteration in cytokine expression profiles following *STK11* suppression. Although PY8119 cells showed a tendency toward enhanced cytokine upregulation, definitive conclusions could not be drawn in the present study due to the absence of ER-positive, mouse-derived breast cancer cell lines available for comparative analysis. Furthermore, the low prevalence of *STK11* mutations in breast cancer remains a significant limitation. According to the MSK-IMPACT® database from cBioPortal, STK11 mutations, including missense, truncating, in-frame shift, splice, and fusion alterations, are detected in only 59 of 3116 (1.9%) breast cancer patients [52]. It is challenging to obtain sufficient *STK11*-mutated breast cancer patients for the study. Therefore, our focus was on STK11-low rather than *STK11*-mutated breast cancers.

We used CRISPR/Cas9 single-guide RNA to knock out the *Stk11* gene in mouse breast cancer cell lines. However, the off-target effects in the CRISPR/Cas9 system can suppress similar sequences throughout the genome and lead to unexpected phenotypes in knockout clones. Therefore, we used three mouse breast cancer cell lines from different origins of cancer cells and tried to eliminate the off-target effects from the CRISPR/Cas9 system.

Low STK11 expression suppresses AMPK activity and activates the mTOR pathway. Metformin, an AMPK activator, has been shown in a meta-analysis to significantly reduce the incidence of breast cancer, with a relative risk of 0.68 (95% CI: 0.55 − 0.83) [53]. A case report documented a near-complete response in a patient with metastatic breast cancer harboring a somatic *STK11* point mutation following treatment with the mTOR inhibitor everolimus [54]. These reports highlight the potential of synergistic therapeutic strategies that combine AMPK activators or mTOR inhibitors with conventional chemotherapeutic agents for treating STK11-deficient breast cancer. Further preclinical and clinical studies are warranted to validate such combination therapies' efficacy and translational potential.

In conclusion, we found that *Stk11* knockout in breast cancer cells enriches immune-related pathways. A significant increase in Cxcl1 expression was detected in response to *Stk11* suppression. In an orthotopic mouse breast tumor model, *Stk11*-KO cancer cells formed larger tumors than their parental counterparts, and circulating PMN-MDSCs were also expanded in mice-bearing *Stk11*-KO tumors. These findings suggest that STK11 loss promotes an immunosuppressive tumor microenvironment. Therefore, restoring STK11 function or targeting its downstream effectors may contribute to reversing immunosuppressive characteristics and provide a potential therapeutic option for STK11-low cancers.

## Supplementary Information

Below is the link to the electronic supplementary material.

Supplementary file1 (DOCX 285 KB)

## Data Availability

The data generated in the present study may be requested from the corresponding author.

## References

[1] Hao J, Gong Y, Zhang X, Du M, Wang H, Guo G, Zhou M, Tian T, Rong H (2025) Global burden of breast cancer and application of patient-reported outcomes in clinical trials: a systematic analysis based on the global burden of disease study 2021 and WHO international clinical trial register database. Front Oncol 15:1557080. 10.3389/fonc.2025.1557080 40575152 10.3389/fonc.2025.1557080 PMC12198119

[2] Wang YY, Wang ZH (2025) Temporal trends in breast cancer treatment patterns and survival: a comprehensive analysis of the SEER database from 1990 to 2017. Curr Probl Surg 69:101795. 10.1016/j.cpsurg.2025.101795 40716853 10.1016/j.cpsurg.2025.101795

[3] Zhong Q, Zhao Y, Zeng L (2025) The clinical effect of breast conservation surgery and modified radical mastectomy on breast cancer: a systematic review and meta-analysis. Curr Probl Surg 68:101772. 10.1016/j.cpsurg.2025.101772 40500032 10.1016/j.cpsurg.2025.101772

[4] Dai Y, Jiang J, Liang P, Yu X, Han Z, Liu F, Tan S, Bi M, Wu C, Cai Q, Li J, Yu J (2024) Percutaneous microwave ablation: a viable local therapy for breast cancer involving the skin/nipple-areola complex? Curr Probl Surg 61(6):101483. 10.1016/j.cpsurg.2024.101483 38823890 10.1016/j.cpsurg.2024.101483

[5] Li X, Peng B, Li J, Tian M, He L (2023) Unleashing breast cancer progression: miR-455-5p’s targeting of SOCS3 drives proliferation, migration, and invasion. Protein Pept Lett 30(12):992–1000. 10.2174/0109298665245603231106050224 38013437 10.2174/0109298665245603231106050224

[6] Bai X, Han G, Li F, Li W, Bu P, Zhang H, Xie J (2023) (R)-9bMS inhibited the protein synthesis and autophagy of triple negative breast cancer cells via regulating miR-4660/mTOR axis. Protein Pept Lett 30(4):295–303. 10.2174/0929866530666230302150750 36861798 10.2174/0929866530666230302150750

[7] Torun V, Degerli E, Cansaran-Duman D (2024) Revealing the molecular signatures of miR-185-5p on breast cancer cells using proteomic analysis. Protein Pept Lett 31(9):681–695. 10.2174/0109298665322427240906060626 39323334 10.2174/0109298665322427240906060626

[8] Talebi M, Farkhondeh T, Harifi-Mood MS, Talebi M, Samarghandian S (2023) Mechanistic features and therapeutic implications related to the miRNAs and Wnt signaling regulatory in breast cancer. Curr Mol Pharmacol 16(5):530–541. 10.2174/1874467216666221017122105 36263474 10.2174/1874467216666221017122105

[9] Hussain MS, Agrawal M, Shaikh NK, Saraswat N, Bahl G, Maqbool Bhat M, Khurana N, Bisht AS, Tufail M, Kumar R (2024) Beyond the genome: deciphering the role of MALAT1 in breast cancer progression. Curr Genomics 25(5):343–357. 10.2174/0113892029305656240503045154 39323624 10.2174/0113892029305656240503045154 PMC11420562

[10] Cursano G, Frigo E, Sajjadi E, Ivanova M, Venetis K, Guerini-Rocco E, Criscitiello C, Curigliano G, Fusco N (2023) Trop-2 as an actionable biomarker in breast cancer. Curr Genomics 24(3):129–131. 10.2174/1389202924666230726112233 38178982 10.2174/1389202924666230726112233 PMC10761338

[11] Schmid P, Cortes J, Dent R, McArthur H, Pusztai L, Kümmel S, Denkert C, Park YH, Hui R, Harbeck N, Takahashi M, Im SA, Untch M, Fasching PA, Mouret-Reynier MA, Foukakis T, Ferreira M, Cardoso F, Zhou X, Karantza V, Tryfonidis K, Aktan G, O’Shaughnessy J, KEYNOTE-522 Investigators (2024) Overall survival with pembrolizumab in early-stage triple-negative breast cancer. N Engl J Med 391(21):1981–1991. 10.1056/NEJMoa2409932 39282906 10.1056/NEJMoa2409932

[12] Araghi M, Gharebakhshi F, Faramarzi F, Mafi A, Mousavi T, Alimohammadi M, Soleimantabar H (2025) Efficacy and safety of pembrolizumab monotherapy or combined therapy in patients with metastatic triple-negative breast cancer: a systematic review and meta-analysis of randomized controlled trials. Curr Gene Ther 25(1):72–88. 10.2174/0115665232283880240301035621 39468438 10.2174/0115665232283880240301035621

[13] O’Farrell J, Lapp C, Kuznia H, Afzal MZ (2025) The role of immunotherapy and immune modulators in hormone-positive breast cancer: implications for localized and metastatic disease. J Clin Med 14(12):4322. 10.3390/jcm14124322 40566067 10.3390/jcm14124322 PMC12194621

[14] Imani S, Farghadani R, Roozitalab G, Maghsoudloo M, Emadi M, Moradi A, Abedi B, Jabbarzadeh Kaboli P (2025) Reprogramming the breast tumor immune microenvironment: cold-to-hot transition for enhanced immunotherapy. J Exp Clin Cancer Res 44(1):131. 10.1186/s13046-025-03394-8 40281554 10.1186/s13046-025-03394-8 PMC12032666

[15] Catalano M, Iannone LF, Nesi G, Nobili S, Mini E, Roviello G (2023) Immunotherapy-related biomarkers: confirmations and uncertainties. Crit Rev Oncol Hematol 192:104135. 10.1016/j.critrevonc.2023.104135 37717881 10.1016/j.critrevonc.2023.104135

[16] Qiu J, Zhou T, Wang D, Hong W, Qian D, Meng X, Liu X (2023) Pan-cancer analysis identifies AIMP2 as a potential biomarker for breast cancer. Curr Genomics 24(5):307–329. 10.2174/0113892029255941231014142050 38235352 10.2174/0113892029255941231014142050 PMC10790333

[17] Hezel AF, Bardeesy N (2008) LKB1; linking cell structure and tumor suppression. Oncogene 27(55):6908–6919. 10.1038/onc.2008.342 19029933 10.1038/onc.2008.342

[18] Krishnamurthy N, Goodman AM, Barkauskas DA, Kurzrock R (2021) *STK11* alterations in the pan-cancer setting: prognostic and therapeutic implications. Eur J Cancer 148:215–229. 10.1016/j.ejca.2021.01.050 33744718 10.1016/j.ejca.2021.01.050 PMC10344467

[19] Cantor DJ, Nimeiri H, Horn L, West M, Ben-Shachar R, Huerga I, Patel JD, Aggarwal C (2025) Outcomes following first-line immune checkpoint inhibitors with or without chemotherapy stratified by KRAS mutational status-a real-world analysis in patients with advanced NSCLC. Clin Lung Cancer. 10.1016/j.cllc.2025.05.007 40544018 10.1016/j.cllc.2025.05.007

[20] Li A, Wang Y, Yu Z, Tan Z, He L, Fu S, Shi M, Du W, Luo L, Li Z, Liu J, Zhou Y, Fang W, Yang Y, Zhang L, Hong S (2023) STK11/LKB1-deficient phenotype rather than mutation diminishes immunotherapy efficacy and represents STING/type I interferon/CD8^+^ T-cell dysfunction in NSCLC. J Thorac Oncol 18(12):1714–1730. 10.1016/j.jtho.2023.07.020 37495171 10.1016/j.jtho.2023.07.020

[21] Skoulidis F, Goldberg ME, Greenawalt DM, Hellmann MD, Awad MM, Gainor JF, Schrock AB, Hartmaier RJ, Trabucco SE, Gay L, Ali SM, Elvin JA, Singal G, Ross JS, Fabrizio D, Szabo PM, Chang H, Sasson A, Srinivasan S, Kirov S, Szustakowski J, Vitazka P, Edwards R, Bufill JA, Sharma N, Ou SI, Peled N, Spigel DR, Rizvi H, Aguilar EJ, Carter BW, Erasmus J, Halpenny DF, Plodkowski AJ, Long NM, Nishino M, Denning WL, Galan-Cobo A, Hamdi H, Hirz T, Tong P, Wang J, Rodriguez-Canales J, Villalobos PA, Parra ER, Kalhor N, Sholl LM, Sauter JL, Jungbluth AA, Mino-Kenudson M, Azimi R, Elamin YY, Zhang J, Leonardi GC, Jiang F, Wong KK, Lee JJ, Papadimitrakopoulou VA, Wistuba II, Miller VA, Frampton GM, Wolchok JD, Shaw AT, Jänne PA, Stephens PJ, Rudin CM, Geese WJ, Albacker LA, Heymach JV (2018) *STK11/LKB1* mutations and PD-1 inhibitor resistance in *KRAS*-mutant lung adenocarcinoma. Cancer Discov 8(7):822–835. 10.1158/2159-8290.CD-18-0099 29773717 10.1158/2159-8290.CD-18-0099 PMC6030433

[22] Turnbull C, Achatz MI, Balmaña J, Castro E, Curigliano G, Cybulski C, Domchek SM, Evans DG, Hanson H, Hoogerbrugge N, James PA, Krause A, Nathanson KL, Ngeow Yuen Yie J, Robson M, Tischkowitz M, Westphalen B, Foulkes WD (2025) Breast cancer germline multigene panel testing in mainstream oncology based on clinical-public health utility: ESMO precision oncology working group recommendations. Ann Oncol S0923–7534(25):00172–00173. 10.1016/j.annonc.2025.04.012 10.1016/j.annonc.2025.04.012 40523834

[23] Lipsa A, Kowtal P, Sarin R (2019) Novel germline STK11 variants and breast cancer phenotype identified in an Indian cohort of Peutz-Jeghers syndrome. Hum Mol Genet 28(11):1885–1893. 10.1093/hmg/ddz027 30689838 10.1093/hmg/ddz027

[24] Sivapiragasam A, Ashok Kumar P, Sokol ES, Albacker LA, Killian JK, Ramkissoon SH, Huang RSP, Severson EA, Brown CA, Danziger N, McGregor K, Ross JS (2021) Predictive biomarkers for immune checkpoint inhibitors in metastatic breast cancer. Cancer Med 10(1):53–61. 10.1002/cam4.3550 33314633 10.1002/cam4.3550 PMC7826457

[25] Lee SJ, Lee CH, Choi SH, Ahn SH, Son BH, Lee JW, Yu JH, Kwon NJ, Lee WC, Yang KS, Lee DH, Han DY, Choi MS, Park PS, Lee HK, Kim MS, Lee J, Jeon BH (2017) Evaluation of a novel approach to circulating tumor cell isolation for cancer gene panel analysis in patients with breast cancer. Oncol Lett 13(5):3025–3031. 10.3892/ol.2017.5807 28521409 10.3892/ol.2017.5807 PMC5431305

[26] STK11. My Cancer Genome. Accessed December 6, 2021. https:// bit.ly/3zn7K5s

[27] Koyama S, Akbay EA, Li YY, Aref AR, Skoulidis F, Herter-Sprie GS, Buczkowski KA, Liu Y, Awad MM, Denning WL, Diao L, Wang J, Parra-Cuentas ER, Wistuba II, Soucheray M, Thai T, Asahina H, Kitajima S, Altabef A, Cavanaugh JD, Rhee K, Gao P, Zhang H, Fecci PE, Shimamura T, Hellmann MD, Heymach JV, Hodi FS, Freeman GJ, Barbie DA, Dranoff G, Hammerman PS, Wong KK (2016) *STK11/LKB1* deficiency promotes neutrophil recruitment and proinflammatory cytokine production to suppress T-cell activity in the lung tumor microenvironment. Cancer Res 76(5):999–1008. 10.1158/0008-5472.CAN-15-1439 26833127 10.1158/0008-5472.CAN-15-1439 PMC4775354

[28] Liu Z, Wu B, Shi X, Zhou J, Huang H, Li Z, Yang M (2025) Immune profiling of premalignant lesions in patients with Peutz-Jeghers syndrome. United Eur Gastroenterol J 13(3):338–348. 10.1002/ueg2.12650 10.1002/ueg2.12650 PMC11999043 39174496

[29] McDonald JH (2014) Handbook of biological statistics, 3rd edn. Sparky House Publishing, Baltimore, Maryland

[30] Korbecki J, Bosiacki M, Barczak K, Łagocka R, Brodowska A, Chlubek D, Baranowska-Bosiacka I (2023) Involvement in tumorigenesis and clinical significance of CXCL1 in reproductive cancers: breast cancer, cervical cancer, endometrial cancer, ovarian cancer and prostate cancer. Int J Mol Sci 24(8):7262. 10.3390/ijms24087262 37108425 10.3390/ijms24087262 PMC10139049

[31] De Giglio A, De Biase D, Favorito V, Maloberti T, Di Federico A, Zacchini F, Venturi G, Parisi C, Gustavo Dall’Olio F, Ricciotti I, Gagliano A, Melotti B, Sperandi F, Altimari A, Gruppioni E, Tallini G, Gelsomino F, Montanaro L, Ardizzoni A (2025) STK11 mutations correlate with poor prognosis for advanced NSCLC treated with first-line immunotherapy or chemo-immunotherapy according to KRAS, TP53, KEAP1, and SMARCA4 status. Lung Cancer 199:108058. 10.1016/j.lungcan.2024.108058 39709652 10.1016/j.lungcan.2024.108058

[32] Candido J, Hagemann T (2013) Cancer-related inflammation. J Clin Immunol 33(Suppl 1):S79-84. 10.1007/s10875-012-9847-0 23225204 10.1007/s10875-012-9847-0

[33] Schabath MB, Welsh EA, Fulp WJ, ChenTeer LJK, Thompson ZJ, Engel BE, Xie M, Berglund AE, Creelan BC, Antonia SJ, Gray JE, Eschrich SA, Chen DT, Cress WD, Haura EB, Beg AA (2016) Differential association of STK11 and TP53 with KRAS mutation-associated gene expression, proliferation and immune surveillance in lung adenocarcinoma. Oncogene 35(24):3209–3216 26477306 10.1038/onc.2015.375 PMC4837098

[34] Kitajima S, Asahina H, Chen T, Guo S, Quiceno LG, Cavanaugh JD, Merlino AA, Tange S, Terai H, Kim JW, Wang X, Zhou S, Xu M, Wang S, Zhu Z, Thai TC, Takahashi C, Wang Y, Neve R, Stinson S, Tamayo P, Watanabe H, Kirschmeier PT, Wong KK, Barbie DA (2018) Overcoming resistance to dual innate immune and MEK inhibition downstream of KRAS. Cancer Cell 34(3):439-452.e6. 10.1016/j.ccell.2018.08.009 30205046 10.1016/j.ccell.2018.08.009 PMC6422029

[35] Li NS, Zou JR, Lin H, Ke R, He XL, Xiao L, Huang D, Luo L, Lv N, Luo Z (2016) LKB1/AMPK inhibits TGF-β1 production and the TGF-β signaling pathway in breast cancer cells. Tumour Biol 37(6):8249–8258. 10.1007/s13277-015-4639-9 26718214 10.1007/s13277-015-4639-9 PMC4875963

[36] Peña CG, Nakada Y, Saatcioglu HD, Aloisio GM, Cuevas I, Zhang S, Miller DS, Lea JS, Wong KK, DeBerardinis RJ, Amelio AL, Brekken RA, Castrillon DH (2015) LKB1 loss promotes endometrial cancer progression via CCL2-dependent macrophage recruitment. J Clin Invest 125(11):4063–4076. 10.1172/JCI82152 26413869 10.1172/JCI82152 PMC4639978

[37] Li R, Salehi-Rad R, Crosson W, Momcilovic M, Lim RJ, Ong SL, Huang ZL, Zhang T, Abascal J, Dumitras C, Jing Z, Park SJ, Krysan K, Shackelford DB, Tran LM, Liu B, Dubinett SM (2021) Inhibition of granulocytic myeloid-derived suppressor cells overcomes resistance to immune checkpoint inhibition in LKB1-deficient non-small cell lung cancer. Cancer Res 81(12):3295–3308. 10.1158/0008-5472.CAN-20-3564 33853830 10.1158/0008-5472.CAN-20-3564 PMC8776246

[38] Zhou X, Fang D, Liu H, Ou X, Zhang C, Zhao Z, Zhao S, Peng J, Cai S, He Y, Xu J (2022) Pmn-mdscs accumulation induced by CXCL1 promotes CD8^+^ T cells exhaustion in gastric cancer. Cancer Lett 532:215598. 10.1016/j.canlet.2022.215598 35176418 10.1016/j.canlet.2022.215598

[39] Qin G, Lian J, Huang L, Zhao Q, Liu S, Zhang Z, Chen X, Yue D, Li L, Li F, Wang L, Umansky V, Zhang B, Yang S, Zhang Y (2018) Metformin blocks myeloid-derived suppressor cell accumulation through AMPK-DACH1-CXCL1 axis. Oncoimmunology 7(7):e1442167. 10.1080/2162402X.2018.1442167 29900050 10.1080/2162402X.2018.1442167 PMC5993496

[40] Kumar V, Donthireddy L, Marvel D, Condamine T, Wang F, Lavilla-Alonso S, Hashimoto A, Vonteddu P, Behera R, Goins MA, Mulligan C, Nam B, Hockstein N, Denstman F, Shakamuri S, Speicher DW, Weeraratna AT, Chao T, Vonderheide RH, Languino LR, Ordentlich P, Liu Q, Xu X, Lo A, Puré E, Zhang C, Loboda A, Sepulveda MA, Snyder LA, Gabrilovich DI (2017) Cancer-associated fibroblasts neutralize the anti-tumor effect of CSF1 receptor blockade by inducing PMN-MDSC infiltration of tumors. Cancer Cell 32(5):654-668.e5. 10.1016/j.ccell.2017.10.005 29136508 10.1016/j.ccell.2017.10.005 PMC5827952

[41] Zhou Z, Zhou Q (2025) Immunotherapy resistance in triple-negative breast cancer: molecular mechanisms, tumor microenvironment, and therapeutic implications. Front Oncol 15:1630464. 10.3389/fonc.2025.1630464 40936705 10.3389/fonc.2025.1630464 PMC12420258

[42] Chen HK, Chen YL, Chung WP, Loh ZJ, Lee KT, Hsu HP (2025) Circulating CD3^+^CD8^+^ T lymphocytes as indicators of disease status in patients with early breast cancer. Cancer Med 14(1):e70547. 10.1002/cam4.70547 39749673 10.1002/cam4.70547 PMC11696249

[43] Ohki S, Shibata M, Gonda K, Machida T, Shimura T, Nakamura I, Ohtake T, Koyama Y, Suzuki S, Ohto H, Takenoshita S (2012) Circulating myeloid-derived suppressor cells are increased and correlate to immune suppression, inflammation and hypoproteinemia in patients with cancer. Oncol Rep 28(2):453–458. 10.3892/or.2012.1812 22614133 10.3892/or.2012.1812

[44] Ouzounova M, Lee E, Piranlioglu R, El Andaloussi A, Kolhe R, Demirci MF, Marasco D, Asm I, Chadli A, Hassan KA, Thangaraju M, Zhou G, Arbab AS, Cowell JK, Korkaya H (2017) Monocytic and granulocytic myeloid derived suppressor cells differentially regulate spatiotemporal tumour plasticity during metastatic cascade. Nat Commun 8:14979. 10.1038/ncomms14979 28382931 10.1038/ncomms14979 PMC5384228

[45] Kline ER, Shupe J, Gilbert-Ross M, Zhou W, Marcus AI (2013) LKB1 represses focal adhesion kinase (FAK) signaling via a FAK-LKB1 complex to regulate FAK site maturation and directional persistence. J Biol Chem 288(24):17663–17674. 10.1074/jbc.M112.444620 23637231 10.1074/jbc.M112.444620 PMC3682567

[46] Gu Y, Lin S, Li JL, Nakagawa H, Chen Z, Jin B, Tian L, Ucar DA, Shen H, Lu J, Hochwald SN, Kaye FJ, Wu L (2012) Altered LKB1/CREB-regulated transcription co-activator (CRTC) signaling axis promotes esophageal cancer cell migration and invasion. Oncogene 31(4):469–479. 10.1038/onc.2011.247 21706049 10.1038/onc.2011.247

[47] Roy BC, Kohno T, Iwakawa R, Moriguchi T, Kiyono T, Morishita K, Sanchez-Cespedes M, Akiyama T, Yokota J (2010) Involvement of LKB1 in epithelial-mesenchymal transition (EMT) of human lung cancer cells. Lung Cancer 70:136–145. 10.1016/j.lungcan.2010.02.004 20207041 10.1016/j.lungcan.2010.02.004

[48] Wang J, Zhang K, Wang J, Wu X, Liu X, Li B, Zhu Y, Yu Y, Cheng Q, Hu Z, Guo C, Hu S, Mu B, Tsai CH, Li J, Smith L, Yang L, Liu Q, Chu P, Chang V, Zhang B, Wu M, Jiang X, Yen Y (2015) Underexpression of LKB1 tumor suppressor is associated with enhanced Wnt signaling and malignant characteristics of human intrahepatic cholangiocarcinoma. Oncotarget 6(22):18905–18920. 10.18632/oncotarget.4305 26056085 10.18632/oncotarget.4305 PMC4662463

[49] Wilkinson S, Hou Y, Zoine JT, Saltz J, Zhang C, Chen Z, Cooper LA, Marcus AI (2017) Coordinated cell motility is regulated by a combination of LKB1 farnesylation and kinase activity. Sci Rep 7:40929. 10.1038/srep40929 28102310 10.1038/srep40929 PMC5244416

[50] Granado-Martínez P, Garcia-Ortega S, González-Sánchez E, McGrail K, Selgas R, Grueso J, Gil R, Naldaiz-Gastesi N, Rhodes AC, Hernandez-Losa J, Ferrer B, Canals F, Villanueva J, Méndez O, Espinosa-Gil S, Lizcano JM, Muñoz-Couselo E, García-Patos V, Recio JA (2020) STK11 (LKB1) missense somatic mutant isoforms promote tumor growth, motility and inflammation. Commun Biol 3(1):366. 10.1038/s42003-020-1092-0 32647375 10.1038/s42003-020-1092-0 PMC7347935

[51] de Jong M, Maina T (2010) Of mice and humans: are they the same?–implications in cancer translational research. J Nucl Med 51(4):501–504. 10.2967/jnumed.109.065706 20237033 10.2967/jnumed.109.065706

[52] de Bruijn I, Kundra R, Mastrogiacomo B, Tran TN, Sikina L, Mazor T, Li X, Ochoa A, Zhao G, Lai B, Abeshouse A, Baiceanu D, Ciftci E, Dogrusoz U, Dufilie A, Erkoc Z, Garcia Lara E, Fu Z, Gross B, Haynes C, Heath A, Higgins D, Jagannathan P, Kalletla K, Kumari P, Lindsay J, Lisman A, Leenknegt B, Lukasse P, Madela D, Madupuri R, van Nierop P, Plantalech O, Quach J, Resnick AC, Rodenburg SYA, Satravada BA, Schaeffer F, Sheridan R, Singh J, Sirohi R, Sumer SO, van Hagen S, Wang A, Wilson M, Zhang H, Zhu K, Rusk N, Brown S, Lavery JA, Panageas KS, Rudolph JE, LeNoue-Newton ML, Warner JL, Guo X, Hunter-Zinck H, Yu TV, Pilai S, Nichols C, Gardos SM, Philip J, Kehl KL, Riely GJ, Schrag D, Lee J, Fiandalo MV, Sweeney SM, Pugh TJ, Sander C, Cerami E, Gao J, Schultz N (2023) Analysis and visualization of longitudinal genomic and clinical data from the AACR project GENIE biopharma collaborative in cBioPortal. Cancer Res 83(23):3861–3867. 10.1158/0008-5472.CAN-23-0816 37668528 10.1158/0008-5472.CAN-23-0816 PMC10690089

[53] Rangraze I, Wali AF, El-Tanani M, Patni MA, Rabbani SA, Babiker R, Satyam SM, El-Tanani Y, Rizzo M (2025) Metformin: a dual-role player in cancer treatment and prevention: a comprehensive systematic review and meta-analysis. Medicina (Kaunas) 61(6):1021. 10.3390/medicina61061021 40572709 10.3390/medicina61061021 PMC12194869

[54] Parachoniak CA, Rankin A, Gaffney B, Hartmaier R, Spritz D, Erlich RL, Miller VA, Morosini D, Stephens P, Ross JS, Keech J, Chmielecki J (2017) Exceptional durable response to everolimus in a patient with biphenotypic breast cancer harboring an *STK11* variant. Cold Spring Harb Mol Case Stud 3(5):a000778. 10.1101/mcs.a000778 28550065 10.1101/mcs.a000778 PMC5593157

