# Supplementary material for: Suppression of STK11 induces expansion of polymorphonuclear myeloid-derived suppressive cells and activation of immune signaling in breast cancer
Source: Cancer Immunol Immunother. 2025 Oct 6;74(11):328. doi: 10.1007/s00262-025-04189-8 (PMC12500487; doi:10.1007/s00262-025-04189-8)
Supplement: Supplementary file 1 — Supplementary file1 (DOCX 285 KB) [file 262_2025_4189_MOESM1_ESM.docx]

| **Supplementary Table 1.** Conjoint differential expression genes (DGEs) were identified in the intersection of three breast cancer cell lines (M158, NF639, and PY8119) following *Stk11*-KO cells. This table lists 56 shared DEGs identified by comparing *Stk11*-KO cells with their corresponding parental controls across all three cell lines. Genes were selected based on consistent differential expression and represent potential downstream targets of *Stk11* loss in mouse breast cancer models. Ratio of fold change (*Stk11*-KO vs. parental cells) and *P* value were shown. | | | | | | |
| --- | --- | --- | --- | --- | --- | --- |
|  | M158 | | NF639 | | PY8119 | |
|  | Ratio | *P* value | Ratio | *P* value | Ratio | *P* value |
| Anxa8 | 4.44 | 5.58E-07 | 2.73 | 0.001 | 22.36 | 1.33E-10 |
| Arl11 | 0.13 | 0.003 | 3.45 | 0.012 | 0.27 | 0.002 |
| Atp13a4 | 0.37 | 0.001 | 29.41 | 1.07E-15 | 13.78 | 9.89E-12 |
| AU018091 | 2.61 | 0.016 | 5.00 | 0.001 | 4.79 | 0.020 |
| Bpifb4 | 0.08 | 8.69E-06 | 0.10 | 1.21E-05 | 0.29 | 0.000 |
| C3 | 3.33 | 2.16E-05 | 7.05 | 6.42E-09 | 19.37 | 5.87E-20 |
| Camk2b | 11.20 | 1.04E-05 | 0.09 | 0.000 | 0.14 | 1.05E-08 |
| Capn5 | 2.04 | 0.012 | 2.23 | 0.008 | 0.22 | 0.041 |
| Cdc42ep2 | 6.90 | 0.000 | 2.22 | 0.014 | 3.07 | 0.001 |
| Cdh5 | 8.29 | 5.20E-11 | 16.71 | 1.34E-10 | 31.94 | 8.26E-24 |
| Cdsn | 5.59 | 0.007 | 2.80 | 0.001 | 3.36 | 5.35E-05 |
| Clec4d | 8951.00 | 0.026 | 23.13 | 8.45E-09 | 2.35 | 0.009 |
| Cxcl1 | 3.91 | 3.13E-05 | 2.23 | 0.012 | 24.12 | 4.94E-22 |
| Cxcl5 | 14.29 | 4.36E-17 | 2.08 | 0.013 | 3628.48 | 0.049 |
| Dmd | 43.35 | 2.84E-12 | 0.14 | 0.001 | 23.60 | 8.46E-08 |
| Etv1 | 2.51 | 0.028 | 0.25 | 2.91E-05 | 0.44 | 0.004 |
| Gm10123 | 0.29 | 1.07E-06 | 0.44 | 0.034 | 0.32 | 0.000 |
| Gm24447 | 4.67 | 0.013 | 8.63 | 5.68E-05 | 0.37 | 0.007 |
| Gm26315 | 4.75 | 0.016 | 10.22 | 3.26E-05 | 0.38 | 0.021 |
| Gm28035 | 2.73 | 0.024 | 0.21 | 0.002 | 0.10 | 3.21E-05 |
| Gng2 | 6.65 | 0.016 | 11.08 | 2.56E-05 | 4.54 | 1.08E-06 |
| Gpc1 | 2.01 | 0.019 | 2.46 | 0.003 | 4.03 | 1.50E-06 |
| Gsta4 | 2.20 | 0.008 | 4.13 | 1.04E-05 | 13.31 | 0.010 |
| Has2 | 3.57 | 0.003 | 2.79 | 0.003 | 4.51 | 0.000 |
| Hivep3 | 0.13 | 0.001 | 0.44 | 0.019 | 0.37 | 0.000 |
| Igfbp4 | 2.03 | 0.026 | 2.63 | 0.001 | 7.73 | 8.33E-12 |
| Kcnab2 | 0.43 | 0.026 | 2.01 | 0.026 | 0.37 | 0.003 |
| Lcn2 | 9.60 | 9.76E-15 | 4.60 | 0.044 | 56.03 | 3.26E-21 |
| Mfhas1 | 2.49 | 0.030 | 0.42 | 0.034 | 0.44 | 0.008 |
| Mst1 | 2.70 | 0.038 | 3.70 | 0.000 | 0.48 | 0.022 |
| Nbea | 3.78 | 4.18E-06 | 0.42 | 0.024 | 2.48 | 0.002 |
| Nfkbie | 3.83 | 0.001 | 3.75 | 0.000 | 2.12 | 0.035 |
| Nnt | 3.94 | 4.31E-05 | 0.00 | 7.25E-45 | 0.36 | 0.001 |
| Nod2 | 28.84 | 1.11E-08 | 7155.56 | 8.37E-05 | 4.40 | 3.11E-06 |
| Nos2 | 7763.19 | 1.38E-05 | 19.77 | 1.79E-05 | 62.49 | 7.22E-25 |
| Nsd1 | 0.16 | 6.08E-06 | 12.81 | 4.44E-06 | 306.39 | 5.40E-38 |
| Odaph | 2.67 | 0.012 | 4.37 | 0.015 | 4.83 | 6.68E-06 |
| Pcdhb14 | 5.17 | 0.009 | 0.14 | 0.013 | 4.04 | 6.12E-05 |
| Pcdhb5 | 5.04 | 0.002 | 0.12 | 0.021 | 4.36 | 1.80E-06 |
| Pde4b | 18.24 | 2.63E-19 | 17.76 | 1.21E-13 | 9.00 | 2.74E-12 |
| Pde4d | 4.55 | 3.18E-07 | 2.79 | 0.004 | 363.00 | 2.25E-33 |
| Plat | 3.74 | 3.84E-05 | 4.28 | 2.47E-05 | 3.36 | 4.65E-05 |
| Pmp22 | 6.72 | 7.18E-06 | 5.51 | 3.36E-07 | 3.71 | 1.31E-05 |
| Ptpn13 | 2.44 | 0.003 | 2.10 | 0.012 | 4.92 | 4.91E-07 |
| Sema3c | 5.70 | 0.008 | 22.89 | 1.01E-16 | 79.15 | 4.16E-28 |
| Slc43a2 | 2.00 | 0.001 | 6.79 | 3.12E-10 | 3.28 | 0.000 |
| Slc5a3 | 0.48 | 0.002 | 0.29 | 0.001 | 0.19 | 1.75E-08 |
| Slfn2 | 3.18 | 0.001 | 36.80 | 7.64E-15 | 10.61 | 0.000 |
| Smpd3 | 3.61 | 9.99E-06 | 0.11 | 4.99E-05 | 3.17 | 0.001 |
| Spata13 | 26.83 | 1.28E-06 | 2.73 | 0.008 | 21.99 | 1.07E-14 |
| Stk11 | 0.37 | 6.90E-05 | 0.43 | 0.030 | 0.43 | 0.002 |
| Trp53inp1 | 2.08 | 0.023 | 0.44 | 0.033 | 4.39 | 1.24E-05 |
| Ttc39c | 3.72 | 1.33E-05 | 2.52 | 0.003 | 3.85 | 6.35E-06 |
| Tubb2a | 4.15 | 1.98E-06 | 2.58 | 0.003 | 3.98 | 2.62E-06 |
| Ugt1a6a | 0.20 | 3.39E-05 | 17.83 | 1.62E-11 | 11.58 | 7.34E-05 |
| Ugt1a7c | 8.04E-05 | 5.80E-07 | 4.34 | 0.006 | 18138.27 | 3.99E-12 |

| **Supplementary Table 2**. Differential expression genes (DEGs) with significant and concordant expression changes in *Stk11*-KO cells versus their parental counterparts across three mouse breast cancer cell lines. This table presents DEGs showing statistical significance and a consistent direction of expression change (either upregulation or downregulation) in all three-mouse breast cancer cell lines (M158, NF639, and PY8119) following *Stk11* knockout. These genes represent robust candidates for downstream effectors of Stk11 loss. | | | | | | | |
| --- | --- | --- | --- | --- | --- | --- | --- |
| Gene name | Gene type | M158 | | NF639 | | PY8119 | |
|  |  | Log_2_ (Fold change) | *P* | Log_2_ (Fold change) | *P* | Log_2_ (Fold change) | *P* |
| Inflammation-related genes | | | |  |  |  |  |
| Nod2 | Protein coding | 4.85 | 1.11E-08 | 12.80 | 8.37E-05 | 2.14 | 3.11E-06 |
| Nos2 | Protein coding | 12.92 | 1.38E-05 | 4.30 | 1.79E-05 | 5.97 | 7.22E-25 |
| C3 | Protein coding | 1.74 | 2.16E-05 | 2.82 | 6.42E-09 | 4.28 | 5.87E-20 |
| Cytoskeleton-related gene | | | |  |  |  |  |
| Bpifb4 | Protein coding | -3.73 | 8.69E-06 | -3.31 | 1.21E-05 | -1.80 | 0.000475 |
| Cell adhesion/migration-related genes | | | |  |  |  |  |
| Cdh5 | Protein coding | 3.05 | 5.20E-11 | 4.06 | 1.34E-10 | 5.00 | 8.26E-24 |
| Plat | Protein coding | 1.90 | 3.84E-05 | 2.10 | 2.47E-05 | 1.75 | 4.65E-05 |
| Pmp22 | Protein coding | 2.75 | 7.18E-06 | 2.46 | 3.36E-07 | 1.90 | 1.31E-05 |
| Signal transduction-related gene | | | |  |  |  |  |
| Pde4b | Protein coding | 4.19 | 2.63E-19 | 4.15 | 1.21E-13 | 3.17 | 2.74E-12 |


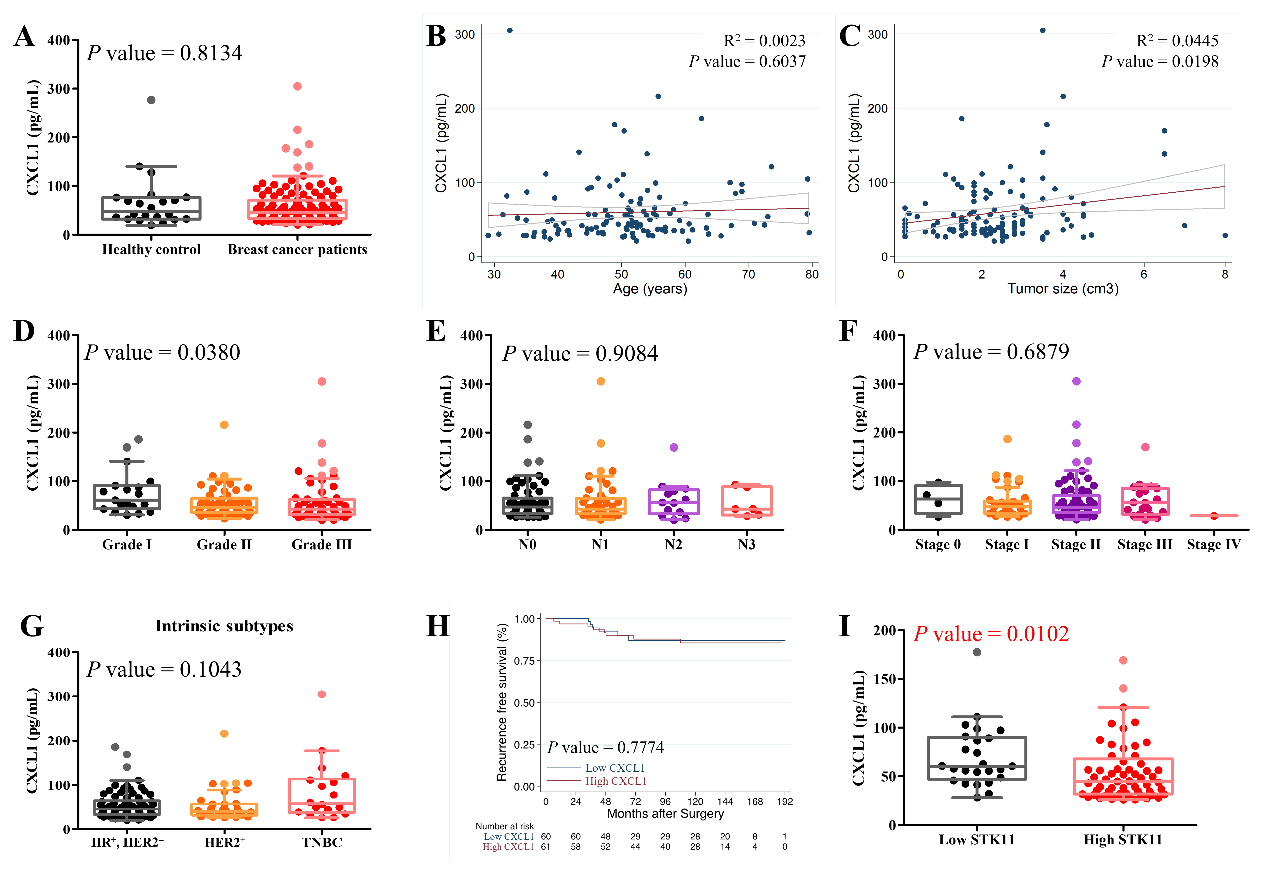


**Supplementary Figure 1.** Plasma level of CXCL1 in breast cancer patients and correlation with clinical characteristics. (A) Comparison of plasma CXCL1 levels between healthy controls and breast cancer patients. (B) Linear correlation between plasma CXCL1 levels and patients’ age. (C) Linear correlation between plasma CXCL1 levels and tumor size. (D) Comparison by histological grade. (E) Comparison by nodal stage. (F) Comparison by TNM stage. (G) Comparison among different molecular subtypes of breast cancer. (H) Kaplan-Meier progression-free survival curve comparing patients with high versus low plasma CXCL1 levels. (I) Correlation between plasma CXCL1 levels and STK11 expression as determined by IHC (referenced in Figure 1).
